# Editorial: Extracellular Vesicles: From Characterization to Treatment

**DOI:** 10.3389/fcell.2022.945529

**Published:** 2022-06-17

**Authors:** Xinlei Li, Jeffrey David Galley

**Affiliations:** ^1^ Center for Clinical and Translational Research, The Abigail Wexner Research Institute at Nationwide Children’s Hospital, Columbus, OH, United States; ^2^ Institute for Behavioral Medicine Research, The Ohio State University Wexner Medical Center, Columbus, OH, United States

**Keywords:** extracellular vesicles, diagnostics, treatment target, disease progression, therapeutics

Extracellular vesicles (EVs) are nanoparticles released from virtually all types of cells, ranging from 50–500 nm in diameter. Some EVs are assembled at and bud from the plasma membrane; these EVs are typically large and called large EVs or ectosomes. Alternatively, some EVs originate from intracellular multivesicular bodies (MVB) and are secreted in bulk through membrane fusion between the MVB and cell membrane. These EVs are relatively small in size and are named small EVs or exosomes. Both EV types have been proven to mediate intercellular communication through the delivery of payloads including proteins, lipids, and nucleic acids, in both physiological and pathological conditions. EV biogenesis represents a snapshot of the status of the producer cells; the yield and composition of EVs can be regulated by different stimuli. Therefore, the EVs circulating in the bloodstream or urine can be used as liquid biopsy tools for the diagnosis or prognosis of different diseases. In addition, upon cargo delivery, EVs released from diseased cells or tissues may lead to the initiation or progression of pathogenesis in target cells or tissues. Thus, EVs under pathophysiological conditions represent potential targets for disease treatment. Additionally, natural or engineered EVs derived from normal differentiated cells or stem cells can serve as potent therapeutics against fibrogenic disorders, inflammatory diseases, congenital syndromes, or cancers (Murphy et al., 2019; Claridge et al., 2021). Collectively, because of these properties, the translational potential of EVs as diagnostics, treatment targets, or therapeutics has attracted considerable attention.

In this Research Topic, there were 12 submissions in total: specifically, three review articles (including one mini review), two brief research reports, one method article, and six original research articles. One major aim of the Research Topic involves new advances in EV biogenesis/characterization, and five reports have contributed new findings to it. Alcohol consumption induces liver injury and elevated EV secretion in alcoholic hepatitis patients (Szabo and Momen-Heravi, 2017). Bala et al. reported in their research article that alcohol promotes exosome biogenesis and release from hepatocytes through modulating Rabs, vesicle-associated membrane proteins, and miR-192. It suggests alcohol imposes a systemic impact on a variety of genes to promote EV biogenesis and secretion. -Omics (proteomics, transcriptomics, or lipidomics) tools are a powerful strategy to characterize EVs payloads and reports herein associate proteome profiling of EVs derived from either physiological or pathological conditions. Lim et al. isolated small and large EVs through differential centrifugation and compared the proteome difference in terms of well-known EV markers, evolutionary conservation, and biological functions. In the nervous system, Torres et al. identified hippocampal neuronal-secreted EVs that carry Wnt, specifically Wnt3a, Wnt5a, and Wnt7a on the EV surface membrane, and then detailed how EVs and Wnt become associated. Differential ultracentrifugation is the most common approach to isolate EVs. However, not all laboratories have access to it. Zoia et al. established two protocols for EV isolation from normal or malaria-infected cells, without requiring ultracentrifugation. The high yield and purity of EVs isolated justify that the two methods could have potential use in resource strapped countries. Within the tumor microenvironment, tumor cells release EVs that have emerged as a vital means of cell communication and immune modulation (Xu et al., 2018). In their mini review, Reed et al. discussed how macrophages and tumor-derived EVs interact, in both pro- and anti-tumor ways.

The capacity of EVs to act as cargo carriers and to have unique surface protein signatures has led to them being utilized as sources of biomarkers, particularly for cancer (Bernard et al., 2019; Logozzi et al., 2019) or studied for their involvement in cancer progression (Liu et al., 2019). Advances herein could completely remodel early-stage disease detection and treatment options. This Research Topic provides such a focus, with two novel and important reports that closely associated EV cargoes with cancers, in many cases employing cutting edge methodologies. Verel-Yilmaz et al. identified ADAM8, a metalloprotease-integrin that is expressed on the EV surface, as well as multiple cargo miRNAs as potential biomarkers for pancreatic ductal adenocarcinoma (PDAC), providing a two-pronged approach that exhibits how EV-based diagnostics can utilize both the surface and the cargo of these nanoparticles. Liquid biopsies for biospecimen collection have become attractive alternatives to more invasive procedures and attempts to combine this with economical and rapid EV collection and characterization is a major research question. Purcell et al. utilized liquid biopsies from patients to identify EV biomarkers for non-small cell lung cancer in both protein and RNA collections, focusing on epidermal growth factor receptor mutations that were detected with Western blot and digital droplet PCR.

EVs can be used in non-cancer diseases as well, and in this Research Topic Wang et al. identified EV-associated miRNA-193a as a urinary biomarker for focal segmental glomerulosclerosis, a nephrotic syndrome. Further, the authors here identified how podocytes were capable of transporting miRNAs, suggesting a method by which the disease could be spread through EV communication. Atherosclerosis is another disease covered in this Research Topic by Hildebrandt et al., wherein the authors were able to identify unique miRNA signatures for multiple types of atherosclerotic types, some of which provided further corroboration based on previous studies. Lastly, Nakao et al. demonstrated how EVs that are involved in lipotoxicity during nonalcoholic steatohepatitis carry damage-associated molecular patterns that may spur disease progression and could potentially be used in diagnostics.

In addition to being used as diagnostic biomarkers, EVs are also being studied in potential treatment applications. Much of the excitement regarding these developments stems from paracrinal activity of EVs, as well as the ability to load EVs with specific cargoes that could then be trafficked to distant sites. Here, two manuscripts look at therapeutic effects that could be leveraged from EVs. Wu et al. present a review on the involvement of EVs in mediating cell death, with a focus on dysfunction due to myocardial infarction. The review covers multiple mechanisms that may be future avenues of EV-based therapeutics, including EVs ability to reduce pyroptosis and abrogate cell death. Amini et al. put forward a review of the relationship between melatonin and EV production/efflux. Melatonin is a pleiotropic molecule produced in the pineal gland that could potentially improve the therapeutic effect of stem cell-derived EVs when used in combination (Sun et al., 2017; Alzahrani, 2019; Chang et al., 2019). They detail how melatonin may work with EVs to reduce inflammation and alter the miRNA cargo of EVs, amongst numerous other interactions.

This Research Topic of EVs, focusing on their characterization and potential uses in diagnostics and therapeutics, represent some of the most exciting work presently being performed in this field. Notably, novel scientific reports on focusing on advances in EVs as therapeutic targets, particularly for anti-tumor treatment, were not covered in this Research Topic and should be a point of emphasis in the future. Going forward, studies focusing on the precise characterization of EV payloads would facilitate the diagnostic application of EVs in liquid biopsy and could potentially lead to the translational use of natural or engineered EVs for disease treatment. In general, we are excited for further development in the EV sub-field of treatment and look forward to these studies reported here reaching new readers as well as for the future analyses that will sprout from results and findings published herein.
